# Supplementary material for: A Humanized Yeast Phenomic Model of Deoxycytidine Kinase to Predict Genetic Buffering of Nucleoside Analog Cytotoxicity
Source: Genes (Basel). 2019 Sep 30;10(10):770. doi: 10.3390/genes10100770 (PMC6826991; doi:10.3390/genes10100770)
Supplement: Supplementary file 1 [file genes-10-00770-s001.zip › Additional_Files/Additional_File7_GOTermHeatmaps.zip!/Additional_File7_GOTermHeatmaps/nuclear_replication_fork.pdf]

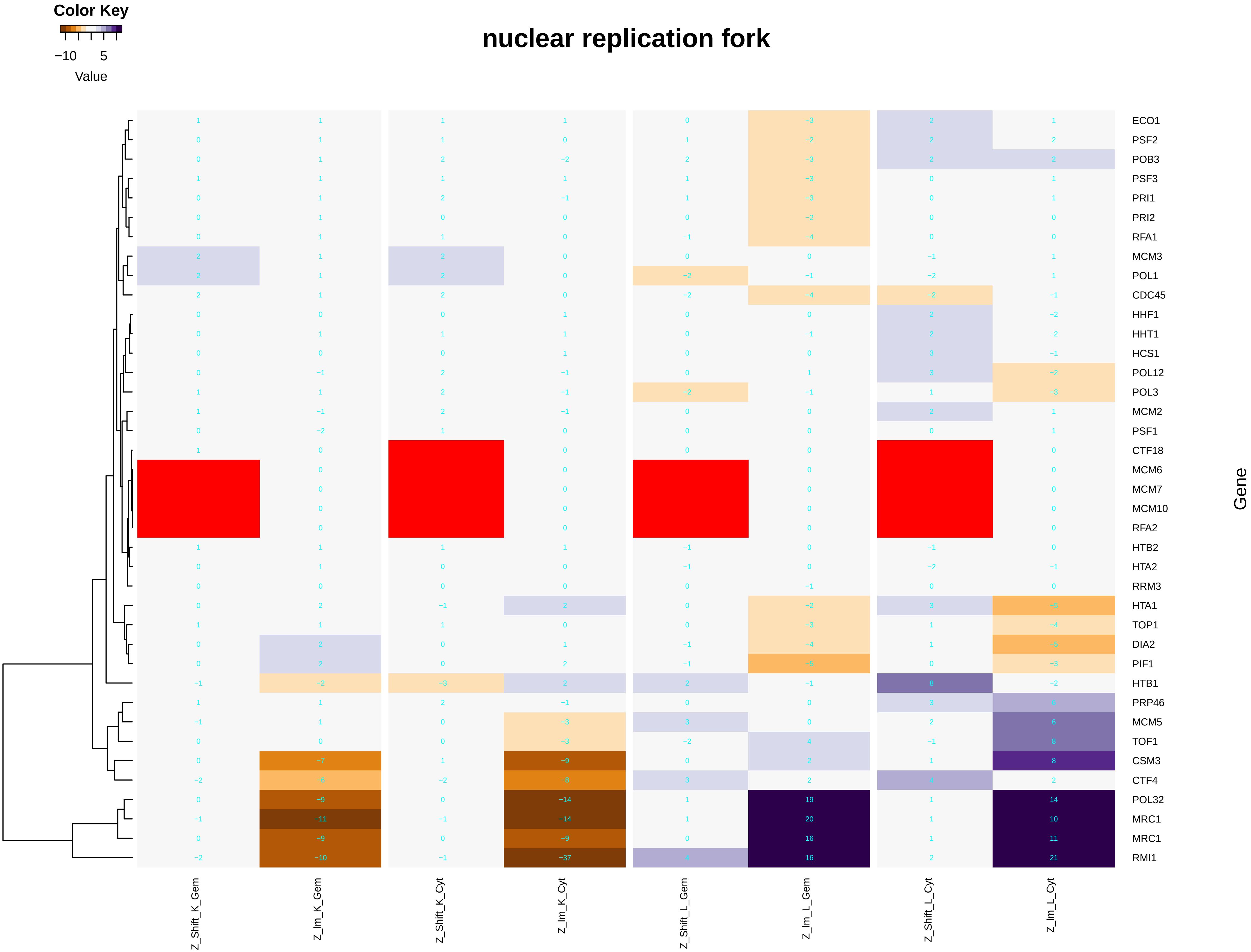
nuclear replication fork
Color Key
−10
5
Value
Gene
ECO1
PSF2
POB3
PSF3
PRI1
PRI2
RFA1
MCM3
POL1
CDC45
HHF1
HHT1
HCS1
POL12
POL3
MCM2
PSF1
CTF18
MCM6
MCM7
MCM10
RFA2
HTB2
HTA2
RRM3
HTA1
TOP1
DIA2
PIF1
HTB1
PRP46
MCM5
TOF1
CSM3
CTF4
POL32
MRC1
MRC1
RMI1
Z_Shift_K_Gem
Z_Im_K_Gem
Z_Shift_K_Cyt
Z_Im_K_Cyt
Z_Shift_L_Gem
Z_Im_L_Gem
Z_Shift_L_Cyt
Z_Im_L_Cyt
